# Voltage Response of a Pyroelectric Detector to a Single Rectangular Optical Radiation Pulse

**DOI:** 10.3390/s22166265

**Published:** 2022-08-20

**Authors:** Andrzej Odon, Anna Szlachta

**Affiliations:** 1 Jan Amos Komenski University of Applied Sciences in Leszno, 64-100 Leszno, Poland; 2 Department of Metrology and Diagnostic Systems, Faculty of Electrical and Computer Engineering, Rzeszow University of Technology, 35-959 Rzeszow, Poland

**Keywords:** mathematical modeling of pyroelectric detector, voltage response of pyroelectric detector

## Abstract

In this paper, a mathematical description of the voltage response of a pyroelectric detector to a single rectangular pulse of optical radiation is presented. Mathematical procedures leading to the derivation of the dependencies describing the detector response for the cases, taking into account the duration of the radiation pulse absorbed by the detector and the relations between values of the electrical and thermal time constants of the detector, are shown in detail. The analytical results are compared and verified by experimental studies carried out using a commercially available pyroelectric detector (model PE10-S-Q) manufactured by the company Ophir. Comparison of the experimentally obtained data with analytical calculations showed good agreement.

## 1. Introduction

Pyroelectric detectors are among the most effective and relatively inexpensive types of uncooled thermal sensors widely used for infrared detection in both civilian and military applications. They are often used in thermal imaging systems, remote temperature measurements, monitoring and measurements of laser radiation parameters, and safety systems. These applications stimulate the permanent development of studies on new pyroelectric materials and structural solutions for detectors [1,2]. Furthermore, studies have been carried out with the aim of improving the mathematical models that describe the properties of pyroelectric detectors [3], methods of performing experimental [4,5] and simulation studies [6] of these detectors, and verification of pyroelectric detector models using advanced optimization algorithms [7,8].

In many applications, the pyroelectric detector is excited by rectangular radiation pulses whose duration is much shorter than their period. If the period of radiation pulses is much longer than the thermal and electrical time constant of the pyroelectric detector, the pyroelectric voltage response *V*(*t*) can be treated as a response to a single radiation pulse. In such cases, all the transient thermoelectric processes of the detector will completely disappear before the next radiation pulse. To evaluate the performance of a pyroelectric detector, it is necessary to find the mathematical description of the voltage response *V*(*t*) of the detector to the rectangular pulse of absorbed radiation with the power *Φ*(*t*). This is particularly important in both the design process of the pyroelectric detector structure and the phase of interpreting the results of its experimental tests. The studies presented in the available literature are focused mainly on the pyroelectric detector response to sine or square wave-modulated radiation, while its response to the radiation pulse is presented only very briefly or even completely omitted. This fact has also been confirmed by the authors of the paper by Efthymiou and Ozanyan [9]. Wheless, Wurtz, and Wells [10] present in detail a methodology to calculate the voltage response of a pyroelectric detector to a radiation signal described by the Dirac pulse, unit step, sinusoidal function, and rectangular signal with 50% duty, but the response to a single rectangular pulse is not taken into account. Shaulov and Simhony [11] analyze the response of a pyroelectric detector to a train of rectangular radiation pulses in steady state. The authors suggest that the mathematical expressions that describe the voltage response of the detector to the periodic impulse train of radiation given in [11] can be transformed into a formula that describes the detector response to a single rectangular radiation pulse; however, this formula is not presented in this article. In fact, only one available work, written by Goodman [12], presents the mathematical expression describing the response of a pyroelectric detector to a single rectangular pulse of radiation absorbed. The expression given in [12] is identical to the results of our calculations (Equation (9)). However, this expression is given in a brief form without any mathematical derivation, and moreover, it refers only to the general case in which the thermal and electric time constants of the detector have different values.

The major goal of the investigation presented in this paper was to find the exact form of mathematical description of the voltage response of a pyroelectric detector to a single rectangular radiation pulse. The equations describing the voltage response were derived for three cases in which the relationships between the thermal and electrical time constant of the detector and the duration of the absorbed radiation pulse were taken into account. Detailed analytical considerations regarding the three above-mentioned cases are important for the practical applications of pyroelectric detectors. For the case described in Section 3.1 and Section 3.2, the analytically determined voltage response of a pyroelectric detector applies to those applications where the power of pulsed radiation sources is monitored. Examples are applications such as radiation power measurements, temperature monitoring [13], intrusion alarms [14], fire alarms, gas spectrometry, and pyroelectric energy harvesting [15]. The analytical considerations presented in Section 3.3 concerning the case when the duration of the radiation pulse is much shorter than the electric and thermal time constant allow determining the voltage response of the pyroelectric detector, the peak value of which is proportional to the energy of the pulsed radiation source. The precisely determined form of the formula (Equation (24)) describing the peak value of the detector response voltage allows for the optimal selection of detector parameters in order to obtain the required sensitivity of the radiation pulse energy measurement.

The analytical results obtained on the basis of the mathematical description of the voltage response of the pyroelectric detector to a single rectangular pulse of radiation proposed in the paper were compared and verified by experimental tests carried out on a commercially available PE10-S-Q pyroelectric detector from Ophir.

## 2. Theoretical Model of Pyroelectric Detector

A pyroelectric detector is essentially a capacitor with capacitance *C_d_.* The dielectric of this capacitor is a thin layer of pyroelectric material of thickness *d* with surface *A* coated with a metallized film. When the pyroelectric detector absorbs radiation power *Φ*(*t*), its temperature changes. As a result of the thermal and electrical processes that take place in the pyroelectric material, an electric charge *q* (*t*) appears on the electrodes of this detector. 

**Figure 1:**
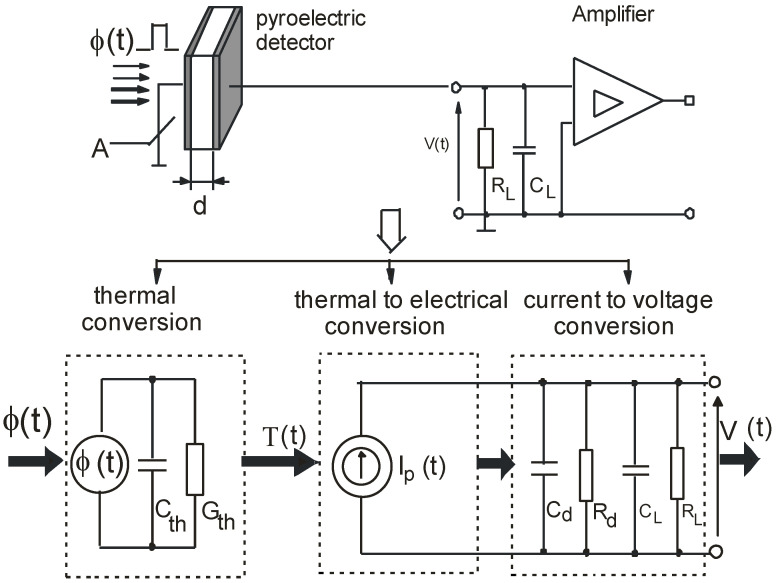
Thermal and electrical equivalent circuit of pyroelectric detector cooperating with amplifier.

In general, the principal properties of pyroelectric detectors are well known. The basic model of the pyroelectric detector, which consists of a mathematical description using linear differential equations and the associated equivalent thermoelectric circuit, was already developed in the 1970s [10,16]. It is worth noting that this model is correct only if it is assumed that the absorbed radiation flux causes a small temperature increase ∆*T*(*t*) of the pyroelectric film, the temperature changes are uniform throughout the volume of this film [9], and the thermal capacity *C_th_* and the thermal conductance *G_th_* of the pyroelectric detector have constant values. Currently, this model, with small adaptations, is still successfully used in studies of pyroelectric detectors, the results of which are presented in numerous articles. It is well established [8] that the conversion process of radiation energy into an electric signal is composed of three stages. The first is the thermal conversion of the radiation flux of the power *Φ*(*t*) incident on the detector surface into a change of the detector temperature ∆*T*(*t*). The second is the thermal to electrical conversion of the temperature changes *T*(*t*) into the electric current of a current source *I_p_*(*t*). The third is the conversion of the current signal *I_p_*(*t*) into a voltage signal *V*(*t*). The equivalent circuit diagram [8] that illustrates the three stages of conversion for a detector that operates in voltage mode is given in Figure 1.

Thermal conversion, thermal-to-electrical conversion, and current-to-voltage conversion are described by the well-known [10] Equations (1)–(3), respectively:

(1) $$C_{th}\frac{d\Delta T\left(t\right)}{dt}+G_{th}\Delta T\left(t\right)=\eta \Phi \left(t\right)$$

(2) $$I_{p}=pA\frac{d\Delta T\left(t\right)}{dt}$$

(3) $$C\frac{dV\left(t\right)}{dt}+\frac{V\left(t\right)}{R}=I_{p}\left(t\right),$$

where *C_th_* is the thermal capacity of the pyroelectric detector, *G_th_* is the thermal conductance of the pyroelectric detector, *η* is the radiation absorption coefficient, *p* is the pyroelectric coefficient, A is the active area of the detector, *C* is the equivalent capacitance for the parallel connected pyroelectric capacitance *C_d_* and the capacitance of the input amplifier *C_L_*: *C* = *C_d_* + *C_L_*, *R* is the equivalent resistance for the parallel connected leakage resistance *R_d_* of the pyroelectric detector and the input amplifier resistance *R_L_*: *R* = *R_d_ R_L_*/(*R_d_* + *R_L_*), and Δ*T*(*t*) is the temperature change of the pyroelectric material.

The equivalent circuit of a pyroelectric detector presented in Figure 1, also called the equivalent circuit of a detector with lumped parameters, and the accompanying mathematical description are often used for the theoretical description of its dynamic properties [5,6,8,9]. This pyroelectric decoder equivalent circuit gives a correct analytical description of pyroelectric detectors with a relatively thin thickness, a relatively small surface area, and a non-complex pyroelectric structure. Due to the use of such assumptions, the spatial temperature distribution of the pyroelectric layer will then be almost identical along all three axes of the pyroelectric, and the mathematical description of heat transfer in the pyroelectric layer can be represented by Equation (1). It is worth noting, however, that the limitations in the application of the simple thermal model are not strictly numerically defined. The experience of many researchers [17,18], including the authors of this publication, shows that the lumped element model of a pyroelectric detector can be used for an analytical description of the dynamic properties of pyroelectric detectors with a thickness of up to 100 µm and an active surface of up to 100 mm^2^. The test results of the PE-10 -SQ pyroelectric detector with a thickness of 100 µm and an active surface of 100 mm^2^ presented in Section 4 showed a good correlation between the experimental and theoretical results. For such geometric parameters of the pyroelectric detector, the spatial temperature distribution will be almost identical along all three axes of the pyroelectric material. As a result, the application of the mathematical description presented in Equations (1)–(3) is fully justified.

The analytical considerations presented in the further sections of this article are based on the lumped-parameter model of a pyroelectric detector and the accompanying Equations (1)–(3), because, according to the authors, both the PVDF detector used to perform the exemplary calculations and the PE-10-S detector (manufactured by Ophir) used in the experimental tests meet the requirements for the application of the lumped-parameter model of the pyroelectric detector.

However, in general, it should be noted that in the case of detectors with a multilayer structure, a relatively large surface area, and thickness of the pyroelectric layer, the analysis should take into account the 3D spatial temperature distribution. Consequently, the mathematical considerations that describe the heat transfer process are complex [19] but provide reliable research results.

In the papers [6] of the coauthor, it was shown that each of the conversion stages illustrated in Figure 1 and described by differential Equations (1)–(3) can be modeled using a Laplace transfer function defined as the ratio of the Laplace transform of the output. 

After performing the appropriate mathematical transformations, presented in detail in [6], one can obtain the Laplace transfer function *G*(*s*) of the pyroelectric detector given by:

(4) $$G\left(s\right)=\frac{V\left(s\right)}{\phi \left(s\right)}=\frac{p\eta }{c^{\prime }dC}\cdot \frac{s\tau_{th}\tau_{e}}{\left(s\tau_{th}+1\right)\left(s\tau_{e}+1\right)}$$

where *V*(*s*) and *Φ*(*s*) represent Laplace transforms of *V*(*t*) and *Φ*(*t*), respectively, *τ_th_* is the thermal time constant of the pyroelectric detector and is defined by the ratio *τ_th_* = *C_th_/G_th_*, *τ_e_ is* the electric time constant of the equivalent circuit shown in Figure 1 and is defined as *τ_e_* = *CR*, and *c’* is the specific volumetric heat of the pyroelectric detector.

The mathematical model of the pyroelectric detector described by Equation (4) in the s-domain is suitable for both analytical and simulation studies of the dynamic properties of this detector using such programming platforms as Matlab-Simulink or LabVIEW.

## 3. Analytical Description of Voltage Pyroelectric Response to the Single Rectangular Pulse

### 3.1. The General Analytical Solution

The Laplace transfer function model of the pyroelectric detector described by Equation (4) may be used to determine the voltage response *V*(*s*) of the pyroelectric detector to the radiation signal of power *Φ*(*s*) in the s-domain. Applying the Laplace transform method, we can obtain the following:

(5) $$V(s)=G\left(s\right)\Phi \left(s\right)=\frac{p\eta }{c^{\prime }dC}\cdot \frac{\tau_{th}\tau_{e}}{\left(s\tau_{th}+1\right)\left(s\tau_{e}+1\right)}\cdot \phi \left(s\right),$$

where *G*(*s*) is the Laplace transfer function of the pyroelectric detector described in Equation (4).

Thus, the voltage response *V*(*s*) to a single rectangular pulse with duration *t_i_* and power amplitude *Φ_m_* is given by:

(6) $$V\left(s\right)=\frac{p\eta }{c^{\prime }dC}\cdot \frac{s\tau_{th}\tau_{e}}{\left(s\tau_{th}+1\right)\left(s\tau_{e}+1\right)}\cdot \phi_{m}\cdot ℒ\left[u\left(t\right)-u\left(t-t_{i}\right)\right],$$

where *Φ_m_* is the power amplitude of radiation, *u*(*t*) is the unit step function, *u*(*t* − *t_i_*) is the shifted unit step function, and £ [*u*(*t*) − *u*(*t* − *t_i_*)] is the Laplace transform of the time domain functions written in square brackets. 

After transformation of the expression £ [*u* (*t*) − *u* (*t* − *t_i_*)] into the Laplace domain, we obtain:

(7) $$\begin{array}{l} V\left(s\right)=\frac{p\eta \phi_{m}}{c^{\prime }dC}\cdot \frac{s\tau_{th}\tau_{e}}{\left(s\tau_{th}+1\right)\left(s\tau_{e}+1\right)}\cdot \left(\frac{1}{s}-\frac{1}{s}\cdot e^{-st_{i}}\right)\\ =K\phi_{m}\cdot \frac{s\tau_{th}\tau_{e}}{\left(s\tau_{th}+1\right)\left(s\tau_{e}+1\right)}\cdot \left(\frac{1}{s}-\frac{1}{s}\cdot e^{-st_{i}}\right) \end{array}$$

where the coefficient *K* is described as:

(8) $$K=\frac{p\eta }{c^{\prime }dC}$$

Finding the inverse Laplace transform of Equation (7), we obtain the expression describing the pyroelectric voltage response *V*(*t*) in the *t* domain:

(9) $$V\left(t\right)=K\cdot \Phi_{m}\cdot \frac{1}{\tau_{e}^{-1}-\tau_{th}^{-1}}\cdot \left(e^{-\frac{t}{\tau_{th}}}-e^{-\frac{t}{\tau_{e}}}\right)\cdot u\left(t\right)\;-K\cdot \Phi_{m}\cdot \frac{1}{\tau_{e}^{-1}-\tau_{th}^{-1}}\cdot \left(e^{-\left(t-t_{i}\right)/\tau_{th}}-e^{-\left(t-t_{i}\right)/\tau_{e}}\right)\cdot u\left(t-t_{i}\right),$$

for the case *τ_e_ ≠ τ_th_*.

Equation (9) describing the pyroelectric voltage response *V*(*t*) is a piecewise function defined by two subfunctions, which are here denoted as voltage signals *V*_1_(*t*) and *V*_2_(*t*). Both the subfunctions *V*_1_(*t*) and *V*_2_(*t*) are applied to a certain time interval of the domain of the main functions *V*(*t*). The voltage signal *V*_1_(*t*) refers to the time interval 0 ≤ *t* ≤ *t_i_* and is described by:

(10) $$V_{1}\left(t\right)=\frac{K\cdot \Phi_{m}}{\tau_{e}^{-1}-\tau_{th}^{-1}}\cdot \left(e^{-t/\tau_{th}}-e^{-t/\tau_{e}}\right)$$

for 0 ≤ *t* ≤ *t_i_* and *τ_e_ ≠ τ_th_*

The voltage signal *V*_2_(*t*) refers to the time *t* under the condition *t* ≥ *t_i_* and is described by:

(11) $$V_{2}\left(t\right)=\frac{K\cdot \Phi_{m}}{\tau_{e}^{-1}-\tau_{th}^{-1}}\cdot \left[e^{-t/\tau_{th}}\left(1-e^{t_{i}/\tau_{th}}\right)-e^{-t/\tau_{e}}\left(1-e^{t_{i}/\tau_{e}}\right)\right],$$

for *t* ≥ *t_i_* and *τ_e_ ≠ τ_th_*.

**Figure 2:**
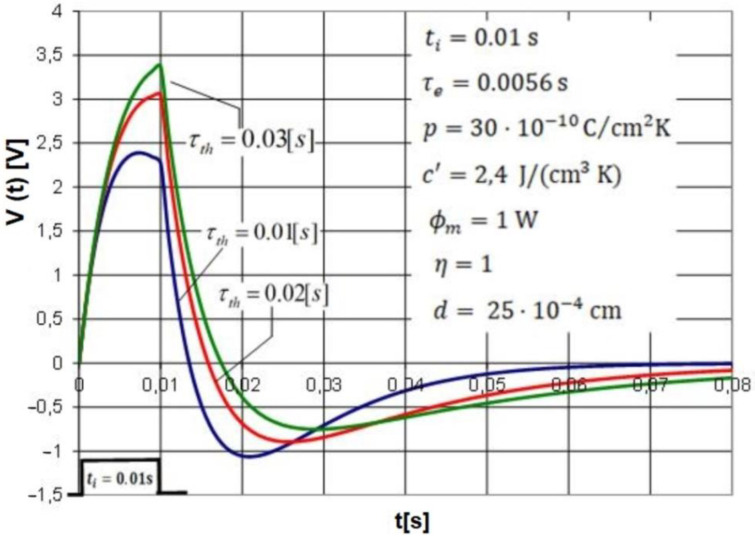
Voltage response *V*(*t*) of PVDF pyroelectric detector to rectangular radiation pulse of duration *t_i_* = 0.01 s for different values of thermal time constant *τ_th_*. The PVDF detector voltage response plots colored blue, red and green are determined for the thermal time constants of *τ_th_ =* 0.01 s, *τ_th_ =* 0.02 s and *τ_th_ =* 0.03 s, respectively. Other parameters of the detector are listed in the figure.

After entering Equations (10) and (11) in the Excel or Matlab program, it is possible to compare the voltage responses *V*(*t*) of the pyroelectric detector for various values of the detector parameters. An example is the study of the influence of the electrical or thermal time constant on the response of a pyroelectric detector. Figure 2 shows graphs of the detector voltage responses to a rectangular radiation pulse of duration *t_i_* = 0.01 s for different values of the thermal time constant. The study was carried out for a prototype of a polyvinylidene fluoride (PVDF) pyroelectric detector designed and made by the author and having well-recognized physical parameters.

Formulas (10) and (11) derived by the authors of the manuscript describe the voltage response of the pyroelectric detector for basically any values of the times *t_i_* of the radial pulse. In particular, these formulas are useful for cases where the value of time *t*_i_ is on the same order as the thermal and electrical time values. In practical applications, a parameter of interest for designers of various types of devices using pyroelectric detectors is the voltage amplitude of the time course of the pyroelectric detector response to excitation by a radiation signal. The family of graphs shown in Figure 2 illustrates one of the many exemplary possibilities of using Formulas (10) and (11) to present the graphical voltage response of the pyroelectric detector obtained for any parameters that characterize the duration *t_i_* and the amplitude *Φ_m_* of the radiation pulse and the physical properties of the pyroelectric detector listed in Figure 2. The graphs shown in Figure 2 provide important information for the detector designer on the effect of the applied values of the time constants *τ_th_* on the sensitivity of the detector excited by a radiation pulse of duration *t_i_* and power *Φ_m_* defined as the ratio of the maximum value of the detector response voltage to the value of the radiated pulse power. The graphs shown in Figure 2 clearly show that increasing the value of the thermal constant gives the desired effect of increasing the sensitivity of the detector.

### 3.2. Analytical Solution for the Special Case of Identical Values of the Thermal and Electric Time Constants

For the special case of *τ_e_ = τ_th_ = τ*, Equations (10) and (11), describing the voltage responses *V*_1_(*t*) and *V*_2_(*t*), respectively, are useless, because after the substitution of *τ_e_ = τ_th_ = τ* into Equations (10) and (11), the results of the calculations have indeterminate forms 0/0. These indeterminate forms of expressions *V*_1_(*t*) and *V*_2_(*t*) can be removed by calculating the limit of the functions *V*_1_(*t*) and *V*_2_(*t)* for *τ_e_ → τ_th_* with the use of the L’Hospital’s rule. As a result, these functions will be transformed into a modified form, *V*_1_*(*t*) and *V*_2_*(*t*). Thus, for 0 ≤ *t* ≤ *t_i_* and *τ_e_ = τ_th_ = τ*, the voltage response *V*_1_*(*t*) is given by:

(12) $$V_{1}^{*}\left(t\right)=\underset{\tau_{e}\to \tau_{th}}{\lim}V_{1}\left(t\right)=\underset{\tau_{e}\to \tau_{th}}{\lim}\frac{K\cdot \Phi_{m\;}}{\tau_{e\;}^{-1}-\tau_{th}^{-1}}\cdot \left(e^{-\frac{t}{\tau_{th}}}-e^{-\frac{t}{\tau_{e}}}\right)\;=\underset{\tau_{e}\to \tau_{th}}{\lim}K\cdot \Phi_{m}\frac{\frac{d}{d\tau_{e}}\left(e^{-\frac{t}{\tau_{th}}}-e^{-\frac{t}{\tau_{e}}}\right)}{\frac{d}{d\tau_{e}}(\tau_{e\;}^{-1}-\tau_{th}^{-1})}\;=\underset{\tau_{e}\to \tau_{th}}{\lim}K\Phi_{m}te^{-t/\tau_{e}}=K\Phi_{m}te^{-t/\tau_{th}}{|}_{\tau_{th}=\tau }=K\Phi_{m}e^{-t/\tau }$$

Similarly, for the special case of *t* ≥ *t_i_* and *τ_e_ = τ_th_ = τ*, the limit of the function *V*_2_*(*t*) for *τ_e_ → τ_th_* is given by:

(13) $$V_{2}^{*}\left(t\right)=\underset{\tau_{e}\to \tau_{th}}{\lim}V_{2}\left(t\right)\;\underset{\tau_{e}\to \tau_{th}}{=\;\lim}\;\frac{K\cdot \Phi_{m}}{\tau_{e\;}^{-1}-\tau_{th}^{-1}}\cdot \left[e^{-t/\tau_{th}}\left(1-e^{t_{i}/\tau_{th}}\right)-e^{-t/\tau_{e}}\left(1-e^{t_{i}/\tau_{e}}\right)\right]\;=\underset{\tau_{e}\to \tau_{th}}{\lim}K\Phi_{m}\frac{\frac{d}{d\tau_{e}}\left(e^{\frac{-t}{\tau_{th}}}-e^{\frac{-t}{\tau_{e}}}-e^{\frac{-\left(t-t_{i}\right)}{\tau_{th}}}+e^{-\frac{-\left(t-t_{i}\right)}{\tau_{e}}}\right)}{\frac{d}{d\tau_{e}}(\tau_{e\;}^{-1}-\tau_{th}^{-1})}\;=K\Phi_{m}\cdot \left[e^{\frac{-t}{\tau_{th}}}-\left(t-t_{i}\right)\cdot e^{\frac{-\left(t-t_{i}\right)}{\tau_{th}}}\right]{|}_{\tau_{th}=\tau }\;=K\Phi_{m}\cdot \left[te^{\frac{-t}{\tau }}-\left(t-t_{i}\right)\cdot e^{\frac{-\left(t-t_{i}\right)}{\tau }}\right]$$

### 3.3. Analytical Solution for a Special Case in Which the Pulse Duration Is Shorter than the Thermal and Electrical Time Constants

In many industrial and medical applications, pulsed radiation sources are used, especially pulse lasers with radiation pulses of short duration. Pyroelectric detectors are the most suitable devices for measuring the power or energy of radiation pulses and have been widely used in techniques for measuring the emission parameters of pulsed lasers for many years. The aim of the considerations presented in this section is to determine the mathematical description of the voltage detector response to a radiation pulse whose shape approximates a rectangular shape and whose duration *t_i_* is much shorter than the electrical and thermal time constants of the detector.

In Section 3.1, it was proven that the first component *V*_1_(*t*) of the pyroelectric voltage response during time interval 0 ≤ *t* ≤ *t_i_* is described by Equation (10). Now, taking into account the assumption that *t_i_ << τ_th_* and *t_i_ << τ_e_*, the voltage response *V_1_*(*t*) will be transformed into the modified mathematical form *V*_1_**(*t*). 

Using a Taylor series expansion for the exponential terms exp(−*t*/*τ_e_*) and exp(−*t*/*τ_th_*) in Equation (10) describing the first component *V*_1_(*t*) of the detector response and assuming that *t_i_* << *τ_th_* and *t_i_* << *τ_e_*, we can approximate these exponential terms by:

(14) $$e^{-t/\tau_{e}}\approx 1-t/\tau_{e}$$

and

(15) $$e^{-t/\tau_{th}}\approx 1-t/\tau_{th}$$

After replacing the exponential terms in Equation (10) by the expressions (14) and (15), we obtain:

(16) $$V_{1}^{**}\left(t\right)=\frac{K\cdot \Phi_{m\;}}{1/\tau_{e}-1/\tau_{th}}\cdot \left(e^{-(t/\tau_{th})}-e^{-\left(t/\tau_{e}\right)}\right)\;\approx \frac{K\cdot \Phi_{m\;}}{1/\tau_{e}-1/\tau_{th}}\cdot \left[\left(1-t/\tau_{th}\right)-\left(1-1/\tau_{e}\right)\right]\;\approx K\cdot \Phi_{m\;}t$$

for 0 ≤ *t* ≤ *t_i_.*

After substituting expression (8) describing coefficient *K* into Equation (16), we obtain the final form of the equation describing the voltage response *V*_1_**(*t*) for the time interval 0 ≤ *t* ≤ *t_i_.*

(17) $$V_{1}^{**}\left(t\right)\approx \frac{p\eta }{c^{\prime }dC}\Phi_{m\;}\cdot t$$

Similarly, taking into account the assumption that *t_i_* << *τ_t__h_* and *t_i_* << *τ_e_*, the second component of the voltage response *V*_2_(*t*) described by Equation (11) will be converted to the modified mathematical form *V*_2_**(*t*). By using a Taylor series expansion for the exponential terms exp(*t_i_/τ_e_*) and exp(*t_i_*/*τ_th_*) in Equation (11) describing the second component *V*_2_(*t*) of the detector response and under the assumption that *t_i_ << τ_th_* and *t_i_ << τ_e_*, we can approximate these exponential terms by:

(18) $$e^{t_{i}/\tau_{e}}\approx 1-t_{i}/\tau_{e,}$$

and

(19) $$e^{t_{i}/\tau_{th}}\approx 1-t_{i}/\tau_{th,}$$

We replace these exponential terms in Equation (11) by expressions (18) and (19), and we obtain:

(20) $$V_{2}^{**}\left(t\right)=\frac{K\cdot \Phi_{m}}{\tau_{e}^{-1}-\tau_{th}^{-1}}\cdot \left[e^{-t/\tau_{th}}\left(1-e^{t_{i}/\tau_{th}}\right)-e^{-t/\tau_{e}}\left(1-e^{t_{i}/\tau_{e}}\right)\right]\;\approx \frac{K\cdot \Phi_{m}}{\tau_{e}^{-1}-\tau_{th}^{-1}}\cdot \left[e^{\frac{-t}{\tau_{th}}}\left(1-1-t_{i}/\tau_{th,}\right)-e^{\frac{-t_{i}}{\tau_{e}}}\left(1-1-t_{i}/\tau_{e,}\right)\right]\;\approx \frac{K\cdot \Phi_{m}\cdot t_{i}}{\tau_{e}^{-1}-\tau_{th}^{-1}}\cdot \left(e^{\frac{-t}{\tau_{e}}}/\tau_{e}-e^{\frac{-t}{\tau_{th}}}/\tau_{th,}\right)$$

After substituting expression (8) describing coefficient *K* into Equation (20), we obtain the final form of the equation that describes the voltage response V_2_**(t) for the time interval *t_i_* ≤ *t:*

(21) $$V_{2}^{**}\left(t\right)\approx \frac{p\eta \cdot \Phi_{m}\cdot t_{i}}{c^{\prime }dC(\tau_{e}^{-1}-\tau_{th}^{-1})}\cdot \left(e^{\frac{-t}{\tau_{e,}}}/\tau_{e}-e^{\frac{-t}{\tau_{th}}}/\tau_{th,}\right)$$

It is worth noting that the energy *E* of a rectangular radiation pulse with duration *t_i_* is described by the dependence:

(22) $$E=\Phi_{m}\cdot t_{i}$$

After replacing the product *Φ_m_ t_i_* in Equation (21) with the energy symbol, *E*, we obtain:

(23) $$V_{2}^{**}\left(t\right)\approx \frac{p\eta \cdot E}{c^{\prime }dC(\tau_{e}^{-1}-\tau_{th}^{-1})}\cdot \left(\frac{e^{\frac{-t}{\tau_{e}}}}{\tau_{e}}-\frac{e^{\frac{-t}{\tau_{th}}}}{\tau_{th,}}\right)$$

for *t* ≥ *t_i_.*

**Figure 3:**
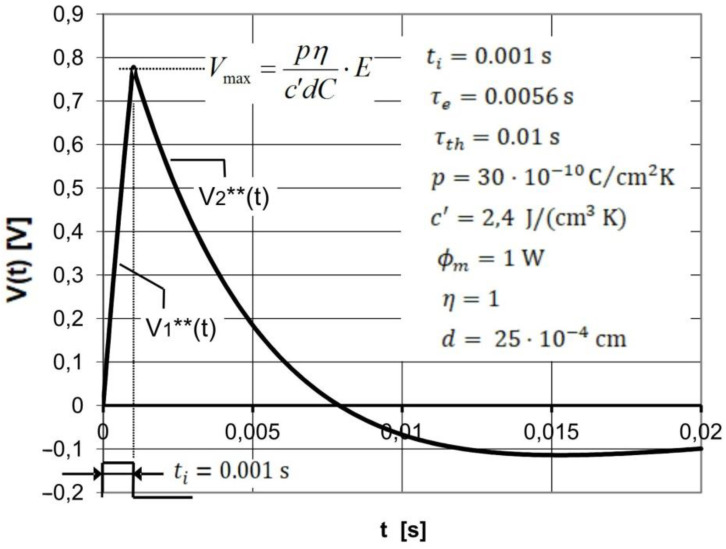
Voltage response *V*(*t*) of the pyroelectric detector to a rectangular radiation pulse of duration *t_i_* significantly shorter than the electrical time constant *τ_e_* and thermal time constant *τ_th_* of the detector. The physical parameters of the detector are listed in the figure. The *V*(*t*) function is a piecewise function defined by two sub-functions *V*_1_**(*t*) and *V*_2_**(*t*) described respectively by the Equations (17) and (21), where each sub-function applies to a different time- interval.

Figure 3 shows the voltage response *V*(*t*) of a pyroelectric detector to a rectangular radiation pulse with a duration *t_i_* much shorter than the electrical time constant *τ_e_* and the thermal time constant *τ_th_* of the detector, which are determined by using Equations (17) and (21).

Equations (17) and (23), which describe the response of the pyroelectric detector as a function of the product power pulse and t of the radiation pulse, are particularly important for the analysis of the performance of the pyroelectric detector used in measurements of pulsed radiation energy. It can be easily shown that the peak voltage *V_max_* of the voltage signals *V*_1_**(*t*) and *V*_2_**(*t*), described respectively by the Equations (17) and (23), occur for time *t* = *t_i_*. After inserting *t* = *t_i_* into Equation (17) or (23), we obtain an equation describing the dependence of the voltage *V_max_* on the energy *E* of pulsed radiation absorbed by the detector:

(24) $$V_{max}=\frac{p\eta }{c^{\prime }dC}\cdot E$$

In catalogue data of pyroelectric detectors intended for energy measurement of radiation pulses, manufacturers usually characterize the detector performance using the voltage sensitivity *R_VJ_* [V/J], which is defined as:

(25) $$R_{VJ}=\frac{V_{max}}{E}$$

The substitution of Equation (24) into Equation (25) leads to a mathematical description of the voltage sensitivity of *R_VJ_* as a function of variables that are directly based on the physical parameters of the pyroelectric detector.

(26) $$R_{VJ}=\frac{V_{max}}{E}=\frac{p\eta }{c^{\prime }dC}$$

## 4. Experimental Results

**Figure 4:**
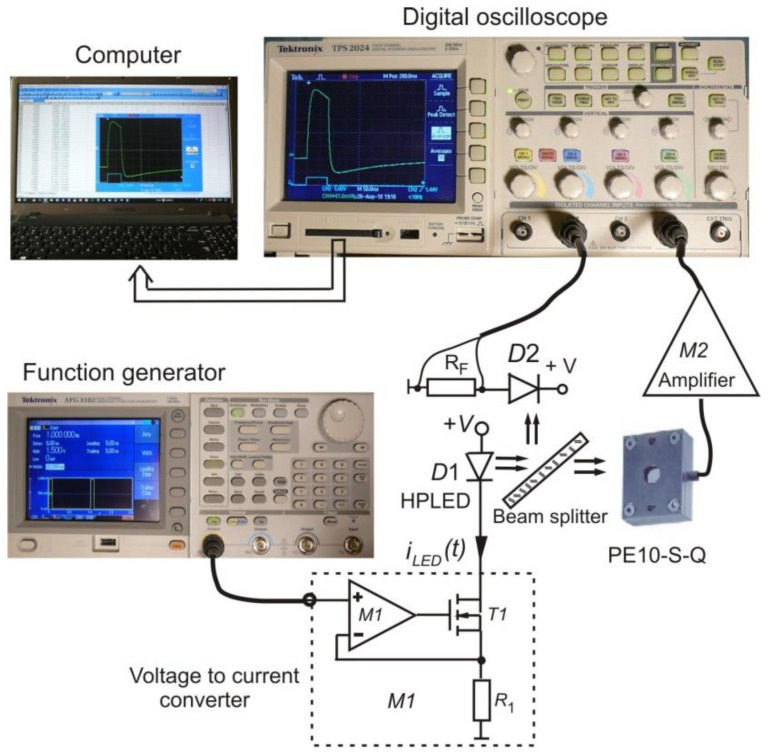
Block scheme of experimental setup employed for measurements of pyroelectric detector response.

An experimental setup, shown in Figure 4, was arranged to display and record the temporal voltage responses of a pyroelectric detector. 

The source of optical radiation was a high-power light-emitting diode (HPLED), model LED660-66-16100, manufactured by Roithner Lasertechnik GMBH. A voltage-to-current converter controlled by a precise functional generator was used as the HPLED driver. The technical solution of the constructed HPLED driver ensured that various shapes of the optical signal emitted by the HPLED would be obtained, including the sinusoidal signal shape, which is necessary for experimental studies of the frequency response of the pyroelectric detector. The optical power of HPLED (D1) was monitored using the photodiode D2. The signals of the output voltage of both the pyroelectric detector and the photodiode circuit were measured using a digital oscilloscope (model TPS2024). A particularly important function of this measurement system was the ability to store data on a memory card in numerical form, which allowed data to easily be transferred to a computer for further processing. The object of the tests was the PE10-S-Q pyroelectric detector with a metallic absorber manufactured by the company Ophir. 

The purpose of the experiments was to compare the voltage response of the PE10-S-Q detector obtained experimentally with the response calculated analytically on the basis of Equations (10) and (11). To determine the analytical response of the pyroelectric detector to a rectangular radiation pulse, it is necessary to insert numerical values of the electrical and thermal time constants *τ_e_* and *τ_th_* into Equations (10) and (11). Unfortunately, the essential parameters of the PE10-S-Q detector, such as the thermal time constant, the capacitance of the detector and the physical parameters of the pyroelectric material used, are not available. For this reason, the experimental studies were divided into two stages. The purpose of the first stage was to determine the values of the thermal and electrical time constants. For this task, the frequency dependence of the responsivity *R_v_* of the PE10-S-Q detector was experimentally determined. Then regression analysis was applied to estimate the values of the electrical and thermal time constants that give the best fit of the data calculated from a regression equation with the experimental data set. The regression equation for the frequency dependence of the responsivity *R_V_* is well known [8] and is given by:

(27) $$R_{V}\left(f\right)=\frac{V_{m}}{\Phi_{m}}\;=\frac{p\eta \tau_{th}\tau_{e}2\pi f}{c^{\prime }dC\sqrt{\left[1+{\left(2\pi f\tau_{th}\right)}^{2}]\cdot [1+{\left(2\pi f\tau_{e}\right)}^{2}\right]}}\;=K\frac{\tau_{th}\tau_{e}2\pi f}{\sqrt{\left[1+{\left(2\pi f\tau_{th}\right)}^{2}]\cdot [1+{\left(2\pi f\tau_{e}\right)}^{2}\right]}}$$

where *K = pη/c’dC* [V/J] can be viewed as constant, *f* is the frequency of sinusoidally modulated optical radiation emitted by HPLED, *Φ_m_* is the amplitude of radiation power absorbed by the detector and *V_m_* is the voltage amplitude of the output detector signal.

**Figure 5:**
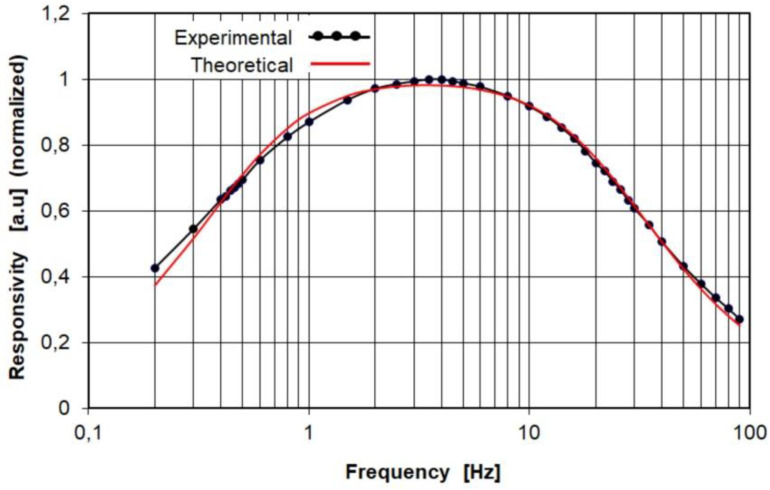
Experimentally (black line with circles) and theoretically (red line) obtained curves illustrating the frequency dependence of the responsivity *R_v_* = *f*(*f*) of the PE10-S-Q pyroelectric detector.

Using the regression procedures based on the least squares method offered by Solver (a Microsoft Excel 2016 add-in program, Redmond, WA, USA), we can find the values of the thermal and electrical time constants of the PE10-S-Q detector. As a result of the best fit of the data calculated from a regression equation with the experimental data set, Solver returned the following parameter values: the thermal time constant *τ_th_* = 0.33 (s) and the electric time constant *τ_e_* = 0.0068 (s). The plots in Figure 5 show the best fit between the experimental and theoretical frequency dependences of the responsivity *R_v_* = *f*(*f*) of the PE10-S-Q pyroelectric detector.

It should be noted that there is a good similarity between the experimentally and theoretically obtained curves illustrating the frequency dependence of the responsivity of the PE10-S-Q pyroelectric detector. This means that the measured electrical and thermal time constants were obtained with good accuracy.

**Figure 6:**
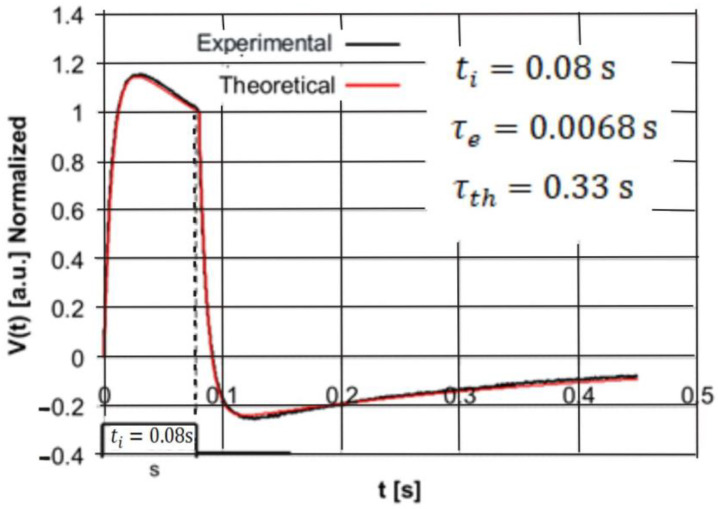
Comparison of the normalized experimental and theoretical voltage responses of the PE10-S-Q detector to the rectangular radiation pulse of duration *t_i_* = 0.08 s.

**Figure 7:**
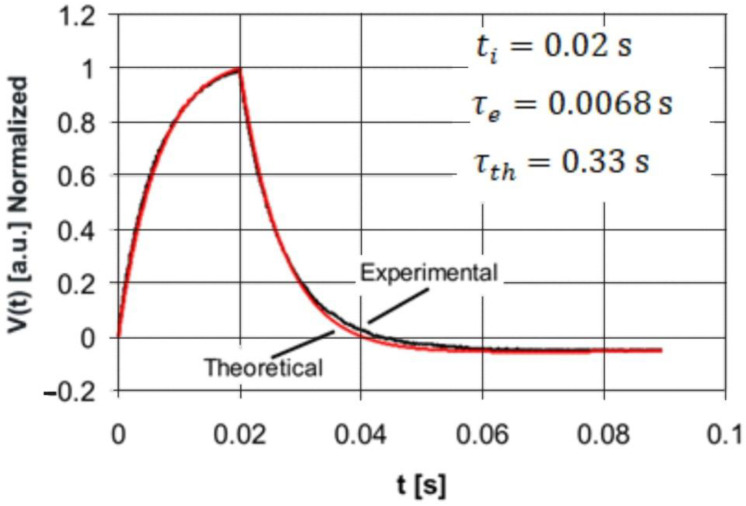
Comparison of the normalized experimental and theoretical voltage responses of the PE10-S-Q detector to the rectangular radiation pulse of duration *t_i_* = 0.02 s.

**Figure 8:**
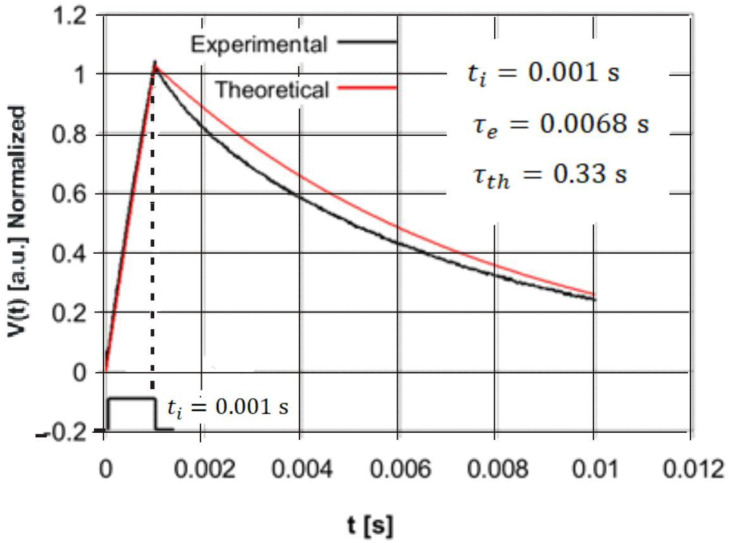
Comparison of the normalized experimental and theoretical voltage responses of the PE10-S-Q detector to the rectangular radiation pulse of duration *t_i_* = 0.001 s.

The purpose of the second stage of the experimental studies was the validation of the analytic Equations (10) and (11) describing the response of the pyroelectric detector to a single rectangular pulse of optical radiation. For this purpose, the PE10-SQ detector was excited by rectangular HPLED radiation pulses with three pulse durations: *t_i_* = 0.08 s, *t_i_* = 0.02 s, and *t_i_* = 0.001 s. As a result of the experiments carried out, the detector response signal was stored on the memory card in the form of a two-column data table containing the values of discrete time instants and the corresponding values of voltage samples. For the same values of discrete time instants, the detector voltage response values were calculated after insertion into Equations (10) and (11) of the values of the electrical and thermal time constants (*τ_th_* = 0.33 s and *τ_e_* = 0.0068 s) and the pulse duration time *t_i_.* Finally, the theoretically calculated data were compared with those obtained experimentally using a normalization procedure and are presented in the form of plots on the same coordinate system in Figure 6, Figure 7 and Figure 8.

A comparison of the plots presented in Figure 6 and Figure 7 shows very good agreement between the study results obtained experimentally and theoretically. Thus, the obtained results of the experiments illustrated in Figure 6 and Figure 7 validate the analytically obtained expressions described by means of Equations (10) and (11) very well. 

The studies whose results are presented in Figure 8 refer to a case in which the duration of the radiation pulse is significantly smaller than the electric and thermal time constants. As already mentioned in Section 3.3, such a requirement occurs in the case of pyroelectric detectors intended to measure the energy of pulsed radiation sources. Figure 8 shows very good agreement between the experimental and theoretical voltage responses of the detector in the time interval of 0 ≤ *t* ≤ *t_i_*. It can be seen that in this time interval, the voltage response of the detector is described by a linear function, which is consistent with Equation (17). It is worth noting that the linear dependence of the detector voltage response to the absorbed rectangular pulse of radiation versus time indicates that the heating of the pyroelectric material occurs adiabatically, as a result of which the peak voltage value is proportional to the energy of the absorbed radiation pulse (see Equation (24)). In the case of the further part of the plots shown in Figure 8 for the time *t* ≥ *t_i_*, a slight discrepancy between the theoretical and experimental plots is noticeable. It seems that this discrepancy is relatively small and does not exclude the use of a mathematical description of the theoretical detector voltage response presented by means of Equation (11) or its approximate form described by Equation (21). 

The time course of this graph shows that the discharge of the pyroelectric detector capacitor is faster than the discharge resulting from the theoretical description. The reasons for this small discrepancy may be due to such causes as: charge leakage caused by imperfect isolation of the output signal transmission path of the pyroelectric detector, the influence of polarization currents of the operational amplifier flowing through the pyroelectric detector capacitor and causing an additional component of the discharge current of this capacitor, non-equilibrium voltage drift (less likely) and other currently undetermined causes.

## 5. Conclusions

The main purpose of the studies described in this paper was to determine the close relationship describing the voltage response of the pyroelectric detector to a single rectangular radiation pulse. Detailed mathematical procedures leading to the derivation of equations describing the detector response for three cases that take into account the duration of the absorbed radiation pulse and the relations between the values of the electrical and thermal time constants of the detector have been presented. The general case concerned different values of the time constant. In the second case, identical values of the time constant were considered. In the third case, analytical considerations were carried out for a radiation pulse whose duration was much shorter than the electrical and thermal time constants of the detector. The results of the studies were illustrated by graphs and discussed. To validate the analytically obtained equations that describe the voltage response of the pyroelectric detector, experimental studies were conducted to compare the theoretical and experimental results. As a measurement object, a commercially available PE10-S-Q pyroelectric detector produced by the company Ophir was used. A comparison of the experimentally obtained data with analytical calculations showed good agreement, and therefore, the mathematical relationships presented give a reliable mathematical description of the voltage response of the PE10-S-Q pyroelectric detector to a single rectangular radiation pulse.

It should be noted that the experimental tests were carried out on the PE10-S-Q detector, which belongs to the group of so-called uncoated pyroelectric detectors, where the surface of the front electrode does not have a black absorber layer such as carbon-based paint. As mentioned in Section 2, pyroelectric detectors with multilayer structures may require a more complex mathematical model than the one described in Section 2. Thus, in these cases, the compliance of the analytically calculated data based on Equations (10) and (11) with the experimental data may not be satisfactory.

## Data Availability

All calculated and measured data will be provided upon request to the correspondent authors by email with appropriate justification.
